# Traumatic urologic injuries in Ile-Ife, Nigeria

**DOI:** 10.4103/0974-2700.70742

**Published:** 2010

**Authors:** Abdulkadir Ayo Salako, Adewale Oluseye Adisa, Amogu K Eziyi, Oluseyi O Banjo, Tajudeen A Badmus

**Affiliations:** Department of Surgery, Obafemi Awolowo University and Obafemi Awolowo University Teaching Hospitals Complex, Ile-Ife, Nigeria

**Keywords:** Trauma, urologic injuries, Nigeria

## Abstract

**Background:**

In a developing country with limited healthcare resources, traumatic injuries and their management pose a significant challenge to healthcare delivery.

**Aim:**

To highlight the challenges in the management of traumatic urologic injuries in patients in our setting.

**Setting and Design:**

Patients presenting with traumatic injuries to the urinary tract, between January 1996 and December 2005, in a University Teaching Hospital in Southwestern Nigeria were the subjects of this study.

**Patients and Methods:**

Clinical records of patients who had such injuries were reviewed.

**Results:**

Ninety injuries occurred in 86 patients including 77 males and 9 females aged 14–68 years. Fourteen (15.5%) of the injuries involved the kidneys, urinary bladder was involved in 23 (25.6%) and the male urethra in 53 (58.9%) injuries. The mechanisms of injury were road traffic accidents in 52 (60.5%) patients, straddle injuries in 18 (20.9%), trauma to the back in 8 (9.3%), falls from a height in 6 (7.0%) and gunshot injuries in 2 (2.3%) patients. Associated injuries include pelvic fractures in 33 (38.4%) patients, limb bone fractures in 13 (14.1%), intestinal injuries in 12 (13.0%) and spinal injuries in 8 (8.7%) patients. In most patients, diagnosis was made based on clinical suspicion and minimal investigations such as abdominal ultrasound, urethrocystoscopy and/or urethrocystography. The outcome was good in most patients and mortality was recorded in only 2 (2.3%) patients who had concomitant spinal and burns injuries.

**Conclusion:**

Prompt management instituted on clinical suspicion of injuries presents a good outcome in patients in a limited resource setting.

## INTRODUCTION

The entire urinary tract, apart from most parts of the male urethra, is fairly well protected from direct injuries from external forces. In most of the published series, urinary tract involvement accounts for less than 10% of the multiple injuries in trauma patients.[1–3] The presentation and management of this injuries have evolved over the years with advancement in the imaging techniques available to surgeons. Specific management of urinary tract injuries following trauma is now tailored to the grade of injury commonly obtainable from preoperative imaging techniques.[4] Thus, computerized tomographic (CT) axial scan has been recognized as the gold standard in the investigation and grading of renal injuries, while magnetic resonance imaging (MRI) and angiography are more common armaments of the urologists in many developed countries.[5–7]

In Nigeria, as indeed many other developing countries, traumatic urologic injuries are commonly seen in association with road traffic accidents.[8–10] The challenges of diagnosis and management in emergency conditions in a limited resource setting without many necessary facilities can be enormous. This study therefore reviews the pattern of urologic injuries among adult patients seen at the Obafemi Awolowo University Teaching Hospitals Complex (OAUTHC), Ile-Ife, Southwestern Nigeria, over a 10-year period.

## PATIENTS AND METHODS

All adult patients presenting with injuries to the urinary tract at the OAUTHC, between January 1996 and December 2005, were the subjects of this study. Sociodemographic data of the patients, including history of the trauma sustained as well as clinical data regarding the signs and symptoms at initial presentation, were examined. We thereafter reviewed the findings on clinical investigations such as cystoscopy and radiological investigations including abdominal ultrasound, urethrocystography or cystourethrography and CT scan. The dossier of patients’ treatment, outcome and complications was also reviewed. The ureteric injuries recorded within the period of this study were from iatrogenic injuries during gynecologic operations performed in private hospitals before referring to us. They were therefore excluded from this study.

The data were then subjected to descriptive analysis.

## RESULTS

Within the study period, 90 injuries were recorded in 86 patients, with 4 male patients sustaining injuries to two urologic sites at the same time. The majority (77 of 86) were males, while 9 (10.5%) were females. Their age ranged between 14 and 68 years, with a mean age of 32.5 years. The mechanisms of injury were road traffic accidents in 52 (60.5%) patients, straddle injuries in 18 (20.9%), trauma to the back in 8 (9.3%), falls from a height in 6 (7.0%) and gunshot injuries in 2 (2.3%) patients, as shown in Table 1. Associated injuries occurred in 48 (55.8%) patients, including pelvic fractures in 33 (38.4%) patients, limb bone fractures in 13 (15.1%), intestinal injuries in 12 (14.0%) and spinal injuries in 8 (9.3%) patients.

**Table 1:** Mechanisms and sites of urologic injuries

Mechanisms of injury	Kidneys	Urinary bladder	Urethra
Vehicular accidents	4	13	39
Straddle injuries	–	–	18
Trauma to the back	8	–	–
Falls from a height	–	3	3
Gunshot injuries	2	–	–

Renal injuries constituted 15.5% of the total, occurring in eight male and six female patients, whose age ranged between 22 and 68 years, with a mean age of 36.2 years. Blunt trauma to the back led to the injury in eight (57.1%) patients, four (28.6%) had automobile accidents, while two (14.3%) of the patients had gunshot injuries involving the kidneys. Among these patients, 11 (78.6%) presented with total hematuria with 5 (35.7%) already in hypovolemic shock, 4 (28.6%) complained of loin pain alone, while 8 (57.1%) had features of peritonitis on initial assessment. The associated injuries sustained included intestinal perforations in five, spinal injuries in two and limb fractures in three patients.

The urinary bladder was the site of injury in 20 male and 3 female patients whose age ranged between 18 and 42 years, with a mean age of 29.4 years. The mechanisms of injury were blunt trauma from automobile accidents in 13 (56.5%), suspected penetrating bone chip from concomitant pelvic fractures in 7 (30.4%), and 3 (13.0%) others fell from heights. Diagnosis was established based on clinical presentation of symptoms such as hematuria, acute urinary retention and abdominal distension. Cystography and/or cystoscopy were done in all but four patients (those with concomitant urethral injuries), with findings of 5 (21.7%) extraperitoneal bladder ruptures, 6 (26.1%) intraperitoneal ruptures, 6 (26.1%) bladder contusions and a combination of extraperitoneal and intraperitoneal ruptures in 2 (8.7%) patients. Mortality was recorded in one patient with associated spinal injury.

Urethral injuries constituted 58.9% of the total, occurring in 53 males with no occurrence in females. Their age ranged between 21 and 58 years, with a mean of 32.4 years. The majority (39 of 53, 73.6%) resulted from automobile accidents, straddle injuries occurred in 18 (34.0%) and falls from a height in 3 others (5.7%). Bleeding per urethra was the most common presentation occurring in 40 of the 53 patients, while other common presenting symptoms were acute urinary retention in 22 (41.5%) and perineal swellings in 13 (24.5%) patients.

## DISCUSSION

In our patients with renal injuries, diagnoses were established based on the clinical history, findings on physical examination and radiological imaging. Abdominal ultrasound scan (KUB) and Intravenous Urogram (IVU) were done routinely in stable patients on clinical suspicion. CT scan may be preferred as it defines the extent of the renal injury and shows associated injuries to other organs.[5] However, the nonavailability of this imaging technique in our center in the early part of this study (as well as its high cost when it was later installed) limited its routine use in these patients. Renal angiography was not done in any of the patients for lack of the facility. The absence of some of these investigative modalities does not seem to have significantly impaired our clinical diagnosis as well as the definitive treatment in these patients. For instance, functional status of the contralateral kidneys was confirmed intra-operatively by observing urine from the contralateral ureteric orifice via a cystostomy where a preoperative IVU had not been done. Those with associated bowel injury detected at operation had primary repair by the General Surgeons. The five patients with hypvolemic shock, hematuria and peritonitis had immediate exploratory laparotomy and nephrectomy for shattered kidneys and/or renal pedicle injury. The remaining patients with lesser renal injuries were managed conservatively by clinical observation, serial abdominal ultrasound scanning and hematocrit evaluation. Follow-up evaluation of these patients was poor as majority of them (8 of 14) were lost to follow up within 1 year. However, long-term complications of hypertension, renal fistulae or deranged renal functions were not seen in those patients who came for follow up.

This study shows that road traffic accidents account for the majority (about two-thirds) of traumatic urologic injuries in our setting. This is similar to findings of previous studies in Nigeria and some other developing economies,[9–12] in contradistinction to a report from the United Kingdom where more than half of some urinary tract injuries resulted from sporting activities.[13] The use of motor cycles, popularly called *Okada*, as a means of transportation is on the increase in our part of the country due to inadequate public transportation and this is largely responsible for many of the accidents recorded.[14] It is therefore envisaged that ongoing road safety and infrastructure development efforts by our government may significantly reduce the incidence of urinary tract injuries in the near future.

Bladder injuries accounted for a quarter of the total urologic trauma seen during the study period. These patients were diagnosed based on presentation with hematuria, acute urinary retention and pelvic hematoma or a combination of these symptoms. Management was then based on the diagnosis. All extraperitoneal bladder ruptures were managed conservatively with suprapubic cystostomy, urethral catheterization with closed bladder drainage for 2–3 weeks and prophylactic antibiotics, while intraperitoneal bladder ruptures were treated operatively by exploration, debridement of the injured bladder edges and primary repair in two layers with closed tube peritoneal drain insertion. Cystography is done 2–3 weeks post repair to confirm healing, and catheter urine specimen routinely cultured showed post injury Urinary Tract Infection (UTI) in seven patients and they were treated promptly with antibiotics. Follow up of these patients has been uneventful.

The majority of injuries in this study involved the male urethra. The role of early urethrocystography in characterizing urethral injuries has been described by some authors.[15,16] In our hospital, the logistics of performing retrograde urethrogram with a water soluble contrast material in the emergency circumstances limited its routine use in the early period of this study. At the initial period, majority of urethral injuries presenting with urinary retention were managed with initial suprapubic catheterization followed by retrograde urethrocystogram (RUCG). In recent times, we are able to perform emergency RUCG followed by delayed urethral exploration and primary realignment, 10–14 days after injury, which has been shown by studies to be associated with less morbidity in patients with complete posterior urethral injuries.[17,18] Endoscopic realignment commenced in our center after acquisition of endoscopy equipments and four patients with posterior urethral injuries have had endoscopic realignment. In our center, incomplete posterior urethral injury is managed by suprapubic diversion and urethral catheterization for 4 weeks. Similarly, we pass urethral catheters for 2–3 weeks in partial anterior urethral injuries. In all cases, pericatheter urethrogram is routinely done before removal of urethral catheter. In some urologic units across Nigeria, similar experiences with urethral injuries have been reported.[8,9,19]

This study has highlighted the importance of good clinical acumen for the prompt management of patients with urologic injuries in our setting. Being retrospective in nature, this study relies largely on previously entered details of patient care. The shortcoming of this is that the attending physician might not have asked, sought for and/or documented some of the characteristics that influenced the results of this study. Poor record keeping in our setting may also mean that some documentations were not well kept for review. To address these shortcomings, we are presently designing a local protocol for the initial evaluation, management and outcome analysis of these patients.

## CONCLUSION

Prompt management of urologic injuries based on clinical diagnosis and the available investigations produced a good clinical outcome in our setting. Many injuries, however, resulted from road traffic accidents and these may be reduced with improved infrastructures. We also advocate improved follow up of patients to ascertain long-term outcome.
